# Draft Genome Sequences of *Listeria monocytogenes* Serotype 4b Strains 944 and 2993 and Serotype 1/2c Strains 198 and 2932

**DOI:** 10.1128/genomeA.00482-16

**Published:** 2016-06-02

**Authors:** Aidan Casey, Olivia McAuliffe, Edward M. Fox, Dara Leong, Cormac G. M. Gahan, Kieran Jordan

**Affiliations:** a Teagasc Food Research Centre, Moorepark, Fermoy, Co. Cork, Ireland; b CSIRO Animal Food and Health Sciences, Victoria, Australia; c School of Microbiology, University College Cork, Co. Cork, Ireland; d School of Pharmacy, University College Cork, Co. Cork, Ireland

## Abstract

*Listeria monocytogenes* is a foodborne pathogen and the causative agent of listeriosis among humans and animals. The draft genome sequences of *L. monocytogenes* serotype 4b strains 944 and 2993 and serotype 1/2c strains 198 and 2932 are reported here.

## GENOME ANNOUNCEMENT

*Listeria monocytogenes* is a Gram-positive pathogen responsible for the bacterial infection listeriosis. Listeriosis, which is associated with the consumption of contaminated foods, primarily affects the young, the old, and the immunocompromised, who are most susceptible to the infection (1). At least 95% of strains isolated from food and clinical samples are members of the 1/2a, 1/2b, 1/2c, or 4b serotype (2). Four *L. monocytogenes* strains, identified as either 4b or 1/2c, were isolated during screening of foods and food-processing environments. Strains 198 and 944 were isolated from cheese products in Ireland, while strains 2932 and 2993 were isolated from Australian meat and dairy production facilities, respectively. Pulsed-field gel electrophoresis analysis demonstrated that while strains 198 and 2932 were closely related, differing only by a single band, the pulsotypes of strains 944 and 2993 were indistinguishable.

DNA was prepared from each of the isolates using the DNeasy blood and tissue kit (Qiagen, The Netherlands), as per the manufacturer’s instructions. Library preparation and 300-bp paired-end sequencing were performed using the Illumina MiSeq platform. Raw reads were preprocessed to remove adapter sequences and low-quality reads using the Trimmomatic (version 0.22) software (3). *De novo* assembly of strains 944, 2932, and 2993 was performed using the SPAdes genome assembler tool (version 2.5.1) (4), while *de novo* assembly of strain 198 was performed using the DNAStar Lasergene SeqMan NGen software (DNAStar, Inc., Madison, WI). Open reading frames (ORFs) were predicted using RAST (5), with subsequent annotations verified and manually curated using BLASTp (6) and Artemis (7).

The resulting assemblies generated a total of 10, 12, 13, and 9 contigs (all >40× average coverage) for *L. monocytogenes* strains 944, 2932, 2993, and 198, respectively, with maximum contig sizes of 816,575 bp, 640,274 bp, 562,372 bp, and 605,673 bp, and a contig *N*_50_ of 388,885 bp, 482,234 bp, 420,755 bp, and 516,594 bp, respectively. The draft genome sizes varied from 2.85 to 2.99 Mb, all with a G+C content of 37.9%. RAST annotation identified 2,877, 2,829, 2,876, and 2,986 protein-coding genes in strains 944, 2932, 2993, and 198, respectively. In addition, strain 198 contained a plasmid of 58,536 bp. Genome comparisons revealed 157 ORFs in strain 198 that were absent in the closely related strain 2932, many of which had phage-related or unknown functions. Also, strain 198 harbored genes potentially conferring resistance to vancomycin (AOA13_1524c) and fosfomycin (AOA13_1530c), as well as an aminoglycoside 3-*N*-acetyltransferase (AOA13_1536c), previously reported to confer resistance to antibiotics, such as tobramycin, gentamicin, and streptomycin (8). The results suggest that strain 198 in particular may have a considerably higher tolerance to the effects of antibiotic treatment than the other strains, consistent with previous research indicating a high prevalence for antibiotic and antimicrobial resistance genes among *L. monocytogenes* dairy isolates (9).

### Nucleotide sequence accession numbers.

The whole-genome shotgun projects for these *L. monocytogenes* strains have been deposited at DDBJ/EMBL/GenBank under the accession numbers LJPD00000000 (strain 944), LJPE00000000 (strain 2932), LJPF00000000 (strain 2993), and LJOZ00000000 (strain 198). The versions described in this paper are versions LJPD01000000, LJPE01000000, LJPF01000000, and LJOZ01000000, respectively.
